# Controlling behaviour of transparency and absorption in three-coupled multiple quantum wells via spontaneously generated coherence

**DOI:** 10.1038/s41598-024-58818-w

**Published:** 2024-04-08

**Authors:** Rohit Mukherjee, Rohit Hazra, Nitu Borgohain

**Affiliations:** 1 Theoretical Photonics Group, Department of Physics, Sarala Birla University, Ranchi, 835103 India; 2 Department of Humanities and Sciences (H & S), Malla Reddy Engineering College for Women, Secunderabad, 500100 India; 3 Department of Physics, University of Science & Technology Meghalaya, Techno City, Ri-Bhoi, 793101 India

**Keywords:** Optics and photonics, Physics

## Abstract

This article presents a coherent phenomenon called spontaneously generated coherence (SGC) under the regime of electromagnetically induced transparency (EIT) in a three-coupled multiple quantum wells. We demonstrate that the presence of SGC in these quantum wells lead to intriguing modifications in the transparency window within the absorption spectrum. At the same time, modification of the dispersive nature is also demonstrated which enables the feasibility of the system in diverse applications based on light propagation. The absorption and dispersion responses are found to be varied by the individual strength of the first and second control fields in presence as well as in absence of SGC in the EIT regime. The positional shifting of the transparency window and simultaneous modifications in the dispersive profiles by tuning the control field detunings of both the first and second control fields are also revealed. Some absorption and dispersion contours are illustrated for getting better insights into the modifications of the optical responses via SGC. Finally, by manipulating the strength of the SGC parameter, we observe the changes in the respective position of the transparency window and dispersion curve. It is expected that the current investigations will pave novel ways for innovative applications in quantum communications, and fabrication of advanced photonic devices.

## Introduction

It is well known that quantum coherence and interference are basic mechanisms for controlling the optical responses of atomic and solid-state media. Over the past few years, several interesting phenomena induced by laser-driven quantum coherence are identified in atomic as well as semiconductor quantum wells (SQWs) such as electromagnetically induced transparency (EIT)^1,2^, lasing without inversion^3,4^, enhancement of refractive index^5^, coherent population trapping (CPT)^6^, giant nonlinearity^7,8^, electromagnetically induced grating (EIG)^9,10^, and so on. Amongst these, EIT^2^ is one of the flourishing optical phenomenon, which minimizes the linear absorption of the probe under resonance followed by quantum destructive interference, and at the same time modifies dispersion characteristics within the transparency region. In the subsequent years, the studies related to EIT have produced a superior impact on optics research due to its potential applications in nonlinear and quantum optics like controlling group velocity^11^, quantum information processing and computing^12^, optical memory^13^, nonlinear photonic devices, optical buffers, etc. In recent years, the investigation of EIT governed absorption/dispersion has been extended in several semiconductor quantum dots and optical microcavities due to the formation of multiple transparent windows which can be used for the fabrication of novel photonic devices with multiple data transmission channels^14–18^.

Similar to EIT, there is another kind of quantum coherence phenomenon namely spontaneously generated coherence (SGC) which has also received tremendous attention^19,20^. The phenomenon of SGC is a kind of quantum interference effect which occurs via multiple decay paths in atomic gases with non-orthogonal dipole moments. In addition, to observe the SGC effect in atomic media, the requirement of the spontaneous emission from two or more closely spaced energy levels to a single energy level or vice-versa and non-orthogonality of the dipole moment of the pump-probe transition serves as key candidates. However, there have been quite a few investigations reported on the properties of the SGC without closed-spaced degenerate energy levels, but non-orthogonal dipole moments play a pivotal role in observing SGC under EIT domain in atomic as well as other solid-based media^21–23^. In the last few decades, several researchers have studied the feature of the SGC-induced absorption, and dispersion in three and four-level atomic systems using different excitation schemes such as Ʌ-, V- and Ξ-^23^, N-^24^, double $\Lambda$-^25^, inverted Y-type^26^ etc. The studies revealed that under the effect of SGC, the absorption is much reduced and within the EIT window slope of the normal dispersion is steeper. These properties of SGC endow remarkable applications of pulse propagation from superluminal to subluminal regime^27^, giant Kerr nonlinearity with minimum absorption^28^, all-optical switching and solitonic pulse propagation with the development of optical communication^29,30^, entanglement^16^ etc. In 2015, Chen et al*.*^31^ found the unique feature of the optical bistability to multistability via controlling the threshold strength of the SGC in asymmetric double quantum wells. Shortly thereafter, the enhancement of giant Kerr nonlinearity in plasmonic nanocavity was also proposed in another study^25^. Recently, the investigation of the SGC has also been extended to semiconductor quantum dots. For example, Ameri et al. investigated entanglement characteristics of the quantum entropy in a double quantum dot system via SGC^32^. They also discovered that the entropy of the system is enhanced for significant strength of SGC, pump and tunneling fields. Salhi et al.^33^ observed that the transmission within EIG is greatly modified in the presence of SGC and the resultant intensity of the grating could be tuned via the controlling fields. Controllable SGC-induced surface plasmon polaritons are also highlighted by Rahman et al.^34^. The impact of an incoherent pumping field on all-optical switching was proposed by Dong et al.^29^. Recently, Anh et al.^35^ investigated analytically the absorption, dispersion, and group index of a five-level atomic media under SGC.

To the best of our knowledge, the investigation of the optical response under SGC is mainly focused on atomic media^19–24,26–30^. However, quite recently a few researchers have reported the impact of SGC in solid based media, especially in SQWs^31^. The unique properties of SQWs are that they possess discrete energy levels and optical response shows high nonlinearities due to large electric dipole moments. In addition, transition energies and symmetries can be created as per requirement by choosing suitable structural materials and dimensions. Investigations on SGC in SQWs are performed in the presence of a single probe and a control field (by considering generic three-level systems like Ʌ, V- and Ξ- or ladder-type systems). However, the presence of an additional control field triggers the pathways for coherence generation and hence enhances the optical properties of the medium as a whole. In this article, we consider a four-level Y-type system which is formed by a combination of two three-level ladder-type systems coupled with a single probe and two control fields. The primary advantage of our chosen system is the presence of an additional control field which manifests the enhancement of the coherent process as well as provides additional flexibility in controlling and manipulating quantum states via SGC, pivotal to applications to quantum computing and quantum communication. Motivated by the current developments and applications perspective, we investigate the effect of the SGC on transparency and absorption under the EIT regime in asymmetric multiple quantum wells (MQWs). The results are obtained by solving coupled Maxwell-Bloch equations numerically and then solving it in a steady state.

In this communication, we present the physical model of the asymmetric MQWs and coupled Maxwell Bloch equations for respective probe and control fields. The numerical results of the absorption and dispersion are highlighted, where the SGC on absorption and dispersion of the medium are investigated extensively under the effects of different physical parameters of the applied fields.

## Model

**Figure 1:**
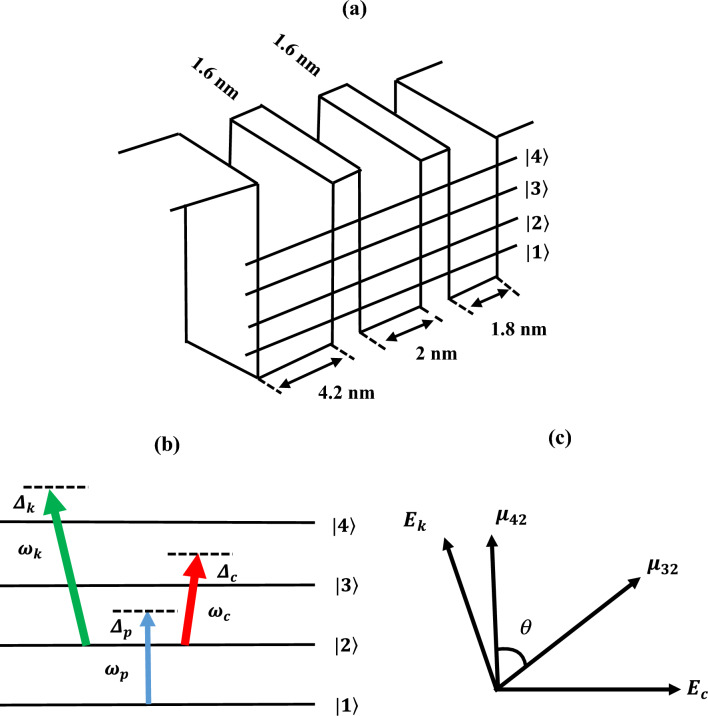
(**a**) Schematic diagram of a single period of the three-coupled MQW nanostructures, (**b**) Y-type excitation scheme with interacting probe $\omega_{p} ,$ and control $\omega_{c} ,\omega_{k}$ fields. (**c**) Direction of the polarization vectors and dipole moment matrix elements for SGC phenomenon.

The quantum system under consideration along with its excitation scheme and polarization directions are presented in Fig. 1. Specifically, we consider 40 periods of three-coupled asymmetric multiple quantum wells (MQWs) with four energy subbands $|1\rangle ,|2\rangle ,|3\rangle ,$ and $|4\rangle$ as shown in Fig. 1a, where all the dipole transitions are allowed. This model of the MQWs was designed by Sirtori et al*.*^36^ in 1992, where they considered each period of the MQWs that consist of $GaInAs$ wells of thickness 4.2, 2.0, and 1.8 nm separated by 1.6 nm $AlInAs$ barriers. The sample considered here, the transition energies are $151, 270, 386,$ and 506 meV respectively. In the following study, we assume that MQWs are designed with low doping concentration, such that the effect arising from electron–electron interaction is negligible. To investigate the nature of absorption and dispersion, we consider a weak probe field of angular frequency ${\omega }_{p}$ applied from energy subbands $\left|1\rangle \right.$ to $\left|2\rangle \right..$ The quantum well system also interacts with two much stronger control laser fields of angular frequencies ${\omega }_{c}$ and ${\omega }_{k}$ applied from subbands $\left|2\rangle \right.$ to $\left|3\rangle \right.$ and $\left|2\rangle \right.$ to $\left|4\rangle \right.$, respectively. For the investigation of the SGC, total interacting driving electric fields in an asymmetric MQWs can be written as,

1 $$E = \left\{ {e^{{ - i\omega_{p} t}} E_{p} + e^{{ - i\omega_{c} t}} E_{c} + e^{{ - i\omega_{k} t}} E_{k} + c.c.} \right\},$$

where $E_{l} \left( {l = p, c, k} \right)$ is the amplitude of the probe, and control fields. Therefore, under the rotating wave and dipole approximations total semi-classical Hamiltonian of the system can be written as,

2 $$\hat{H} = \mathop \sum \limits_{i = 1}^{4} \hbar \omega_{i} \left| i\rangle \langle\right.\left. i \right| - \hbar \left\{ {\Omega_{p} e^{{ - i\omega_{p} t}} \left| 2\rangle \right.\langle\left. 1 \right| + \Omega_{c} e^{{ - i\omega_{c} t}} \left| 3\rangle \right.\left. \langle2 \right| + \Omega_{k} e^{{ - i\omega_{k} t}} \left| 4\rangle \right.\left. \langle2 \right| + h.c.} \right\},$$

here $h.c.$ denote Hermitian conjugate; $\Omega_{p} , \Omega_{c} ,$ and $\Omega_{k}$ are respectively Rabi-frequencies of the probe and control fields which are defined as, $\Omega_{p} = \frac{{\mu_{21} E_{p} }}{\hbar }$, $\Omega_{c} = \frac{{\mu_{32} E_{c} }}{\hbar }$, and $\Omega_{k} = \frac{{\mu_{42} E_{k} }}{\hbar }$, respectively. $\mu_{ij}$ represent the laser-driven inter-subband transition dipole moment matrix element from $\left| i \right. \to \left| j \right.$ transition. For this study, one important point to be noted is that, in our physical model the dipole moment matrix element $\mu_{ij}$ (expressed as $\mu_{ij} = \mathop{\mu_{ij} }\limits^{\rightharpoonup} .\hat{e}_{l} \left( {i,j = 1 - 4} \right)$)^31^ for respective transition that arises from the quantum mechanical treatment of the perturbed Hamiltonian and it is connected with the driving electric fields described in Eq. (1) via Rabi-frequencies of the probe, and control fields. Here, $\hat{e}_{l} \left( {l = p, c, k} \right)$ is the unit vector along the polarization direction of the optical field. The orientation of the electric dipole moments $\mu_{32}$ and $\mu_{42}$ are shown in subplot 1(c), which ensures the non-orthogonal component of the dipole moments and necessary criteria for the existence of the SGC phenomenon. Although Eq. (1) is not fully valid for studying the EIT and related coherence effects via Maxwell-Bloch formalism under the well known rotating wave approximation and this equation can be useful for the study of nonlinear evolution of the pulse propagation, dispersion, saturation, amplification effect and ultrafast pulse excitation of the short pulses propagating in different systems^37,38^. Usually the correct treatment of EIT effect and other coherent light-matter interaction is best done via invoking the full Maxwell-Bloch models^39^. However, in this article we are considering a simple theoretical model based on rotating wave approximation which simplifies the dynamics of light-matter interaction by neglecting rapidly oscillating terms in the Hamiltonian, which make our mathematical treatment more tractable. In the present study of SGC under EIT regime, rotating wave approximation can quite accurately predict the conditions under which absorption and dispersion spectra appear in medium.

To investigate, the system response of the MQWs, we apply the standard Liouville’s method^26^ which is given by,

3 $$\frac{{\partial \rho_{ij} }}{\partial t} = - \frac{i}{\hbar }\mathop \sum \limits_{m} \left( {\hat{H}_{im} \rho_{mj} - \rho_{im} \hat{H}_{mj} } \right) + L\rho ,$$

where $\rho_{ij}$ is density matrix operator, and $L\rho$ represent a combination of spontaneous radiative decay terms which are added phenomenologically. By employing a similar procedure^26,40^, we obtain a set of density matrix equations as follows:

4 $$\dot{\rho }_{11} = \frac{{{\text{i}}\Omega_{p} }}{2}\rho_{21} - \frac{{{\text{i}}\Omega_{p}^{*} }}{2}\rho_{12} + \Gamma_{21} \rho_{22} ,$$

5 $$\begin{aligned} \dot{\rho }_{22} & = \frac{{{\text{i}}\Omega_{p}^{*} }}{2}\rho_{12} - \frac{{{\text{i}}\Omega_{p} }}{2}\rho_{21} + \frac{{{\text{i}}\Omega_{c} }}{2}\rho_{32} - \frac{{{\text{i}}\Omega_{c}^{*} }}{2}\rho_{23} + \frac{{{\text{i}}\Omega_{k} }}{2}\rho_{42} - \frac{{{\text{i}}\Omega_{k}^{*} }}{2}\rho_{24} \\ & \quad + \Gamma_{42} \rho_{44} + \Gamma_{32} \rho_{33} - \Gamma_{21} \rho_{22} + 2p\sqrt {\Gamma_{32} \Gamma_{42} } \cos \theta \left( {\rho_{43}^{*} + { }\rho_{43} } \right), \\ \end{aligned}$$

6 $$\dot{\rho }_{33} = \frac{{{\text{i}}\Omega_{c}^{*} }}{2}\rho_{23} - \frac{{{\text{i}}\Omega_{c} }}{2}\rho_{32} - p\sqrt {\Gamma_{32} \Gamma_{42} } \cos \theta \left( {\rho_{43}^{*} + { }\rho_{43} } \right) - \Gamma_{32} \rho_{33} ,$$

7 $$\dot{\rho }_{44} = \frac{{{\text{i}}\Omega_{k}^{*} }}{2}\rho_{24} - \frac{{{\text{i}}\Omega_{k} }}{2}\rho_{42} - p\sqrt {\Gamma_{32} \Gamma_{42} } \cos \theta \left( {\rho_{43}^{*} + { }\rho_{43} } \right) - \Gamma_{42} \rho_{44} ,$$

8 $$\dot{\rho }_{13} = - \left\{ {\frac{{\Gamma_{32} }}{2} - {\text{i}}\left( {\Delta_{p} + \Delta_{c} } \right)} \right\}\rho_{13} - \frac{{{\text{i}}\Omega_{c} }}{2}\rho_{12} + { }\frac{{{\text{i}}\Omega_{p} }}{2}\rho_{23} - 2p\sqrt {\Gamma_{32} \Gamma_{42} } \cos \theta \rho_{41}^{*} ,$$

9 $$\dot{\rho }_{21} = - \left\{ {\frac{{\Gamma_{21} }}{2} + {\text{i}}\Delta_{p} } \right\}\rho_{21} + \frac{{{\text{i}}\Omega_{k} }}{2}\rho_{41} - \frac{{{\text{i}}\Omega_{p}^{*} }}{2}\rho_{22} + \frac{{{\text{i}}\Omega_{p}^{*} }}{2}\rho_{11} + \frac{{{\text{i}}\Omega_{c} }}{2}\rho_{13}^{*} ,$$

10 $$\begin{aligned} \dot{\rho }_{23} & = - \left\{ {\frac{{(\Gamma_{32} + \Gamma_{21} )}}{2} - {\text{i}}\Delta_{c} } \right\}\rho_{23} + \frac{{{\text{i}}\Omega_{k} }}{2}\rho_{43} - { }\frac{{i\Omega_{c} }}{2}\rho_{22} \\ & \quad + \frac{{{\text{i}}\Omega_{p}^{*} }}{2}\rho_{13} + \frac{{{\text{i}}\Omega_{c} }}{2}\rho_{33} - 2p\sqrt {\Gamma_{32} \Gamma_{42} } \cos \theta \rho_{42}^{*} , \\ \end{aligned}$$

11 $$\dot{\rho }_{41} = - \left\{ {\frac{{\Gamma_{42} }}{2} + {\text{i}}\Delta_{k} + {\text{i}}\Delta_{p} } \right\}\rho_{41} - \frac{{{\text{i}}\Omega_{p}^{*} }}{2}\rho_{42} + \frac{{{\text{i}}\Omega_{k}^{*} }}{2}\rho_{21} - 2p\sqrt {\Gamma_{32} \Gamma_{42} } \cos \theta \rho_{13}^{*} ,$$

12 $$\begin{aligned} \dot{\rho }_{42} & = - \left\{ {\frac{{(\Gamma_{42} + \Gamma_{21} )}}{2} + {\text{i}}\Delta_{k} } \right\}\rho_{42} - \frac{{{\text{i}}\Omega_{k}^{*} }}{2}\rho_{44} - \frac{{i\Omega_{p} }}{2}\rho_{41} \\ & \quad - \frac{{{\text{i}}\Omega_{c}^{*} }}{2}\rho_{43} + \frac{{{\text{i}}\Omega_{k}^{*} }}{2}\rho_{22} - 2p\sqrt {\Gamma_{32} \Gamma_{42} } \cos \theta \rho_{23}^{*} , \\ \end{aligned}$$

13 $$\begin{aligned} \dot{\rho }_{43} & = - \left\{ {\frac{{(\Gamma_{42} + \Gamma_{32} )}}{2} + {\text{i}}\left( {\Delta_{k} - \Delta_{c} } \right)} \right\}\rho_{43} - { }\frac{{i\Omega_{c} }}{2}\rho_{42} \\ & \quad + \frac{{{\text{i}}\Omega_{k}^{*} }}{2}\rho_{23} - 2p\sqrt {\Gamma_{32} \Gamma_{42} } \cos \theta \left( {\rho_{33} + { }\rho_{44} } \right). \\ \end{aligned}$$

Here, the above equations are constrained by normalization condition $\rho_{11} + \rho_{22} + \rho_{33} + \rho_{44} = 1,$ and $\rho_{ij} = \rho_{ji}^{*} \cdot \Delta_{p} = \omega_{p} - \omega_{21} , \;\Delta_{c} = \omega_{c} - \omega_{32} ,$ and $\Delta_{k} = \omega_{k} - \omega_{42}$ are respectively, detuning the probe and control laser fields. $\Gamma_{21} , \Gamma_{32} ,$ and $\Gamma_{42}$ are respectively, the spontaneous decay rates from subbands $\left| 2 \right. \to \left| 1 \right.,\left| 3 \right. \to \left| {2,} \right.$ and $\left| 4 \right. \to \left| {2.} \right.$ In Eqs. (4)–(13), the term $p\sqrt {\Gamma_{32} \Gamma_{42} } \cos \theta$ signify the result of the cross-coupling between spontaneous emission $\left| 3 \right. \to \left| 2 \right.$, $\left| 4 \right. \to \left| {2,} \right.$ and $\theta$ is the angle between two non-orthogonal dipole moment matrix elements $\mu_{32}$ and $\mu_{42}$ (as shown in Fig. 1c), which stands as a significant factor contributing to the SGC. Here, the physical parameter $p$ characterizes the SGC parameter for producing quantum coherence phenomena in the given system^41^. Therefore, SGC-induced quantum interference is greatest for parallel dipole moments, and it will vanish for perpendicular dipole moments. For observing the SGC phenomenon inside MQWs, we have to take $p = 1,$ and for $p = 0$ the effect of SGC vanishes. Because of the existence of the SGC effect in MQWs, the physical properties of the system are modulated by several parameters of the driving laser fields.

It is well known that, the response of the probe field in MQWs is characterized by linear susceptibility $\chi^{\left( 1 \right)}$. To investigate the effect of SGC behaviour on absorption and dispersion properties of the probe field, we have solved the above set of density matrix Eqs. (4)–(13) numerically under steady-state conditions. Therefore, the linear susceptibility of the probe turned out to be^42^

14 $$\chi^{\left( 1 \right)} = Re \left( {\chi^{\left( 1 \right)} } \right) + i \cdot Im \left( {\chi^{\left( 1 \right)} } \right) = \frac{{N\left| {\mu_{21} } \right|^{2} }}{{\hbar \varepsilon_{0} \Omega_{p} }}\rho_{21} ,$$

where $N$ is the carrier concentration per unit volume of the MQWs. $\hbar ,$ and $\varepsilon_{0}$ are respectively, the reduced Planck’s constant and the permittivity in free space. Here, $Re \left( {\chi^{\left( 1 \right)} } \right)$ and $Im \left( {\chi^{\left( 1 \right)} } \right)$ signify the dispersion and absorption properties of the weak probe field. In Eq. (14), $Im \left( {\chi^{\left( 1 \right)} } \right) < 0$ denotes the gain of the probe field, subsequently, $Im \left( {\chi^{\left( 1 \right)} } \right) > 0$ characterizes the loss of the probe field which could be tuned under the effect of SGC.

Since, we are mainly investigating the properties of laser induced quantum coherence in MQWs and it can found by numerically solving the Maxwell-Bloch equations and determining the probe coherence term ($\rho_{21}$), which thus provides the accurate information about the refractive index $\left[ {n = \sqrt {1 + Re \left( {\chi^{\left( 1 \right)} } \right)} } \right]$ of the medium^43^.

## Results

In this section, we first discuss the optical responses of the Y-type system, both in presence and in absence of SGC by tuning only the first control field. Then, we analyse the responses by switching ‘on’ the second control field and to have a clear visualization of the above features, we illustrate some contour plots. Next, we observe the changes in the absorption and dispersion spectra by manipulating the detunings of the control fields under SGC. Also, the angular dependence of SGC on absorption/dispersion is discussed separately. Finally, we explain the effect of the SGC parameter on the optical responses.

### *Effect of *${\Omega }_{c}$* on SGC*

We begin our studies by investigating the absorption and dispersion spectra under the EIT regime in presence as well as in absence of SGC. In all the cases, the system parameters considered here are as follows^36,42^: $N = 10^{24} \;{\text{m}}^{ - 3}$, $\mu_{21} = 13\;{\text{e{\AA}}} = 20.8 \times 10^{ - 29} \;{\text{Cm}}$, and the decay rates have been normalized as, $\Gamma_{21} = 0.48\gamma ,\; \Gamma_{31} = 0.38\gamma , \;\Gamma_{41} = 0.43\gamma$, where $\gamma = 10\Gamma$ is a normalizing factor with $\Gamma = 10.0 \times 10^{12} \;{\text{s}}^{ - 1}$.

The quantum system parameters of the MQWs can be exactly calculated from different quantum transport modelling approaches reported by several researchers^44–46^. However, if we recall Eq. (2), the parameters of the quantum well system are specifically the electronic energy levels, the Rabi frequencies and angular frequencies of the associated laser fields (one probe and two control fields). The electronic energy levels of the particular MQWs could be computed by solving self-consistently the Schrödinger and Poisson’s equations in the envelope function formalism^47,48^. Since the values of the computed energy levels are readily available^36^, we select those values and the curious readers may look upon Ref.^36^ for further investigations. Other system parameters such as dipole moment matrix elements are taken from the available experimental absorption spectrum of the MQWs^49^. Finally, the system parameters involving the associated Rabi frequencies of the laser fields are taken  ~ $10^{12} \;{\text{s}}^{ - 1}$ [For example, $\mu_{21} \sim { }10^{ - 29} \;{\text{Cm}}$, $E_{p} \sim 10^{7}$ V/m; ${ }\Omega_{p}$ ~ $10^{12} \;{\text{s}}^{ - 1}$], consistent with Ref^50^.

**Figure 2:**
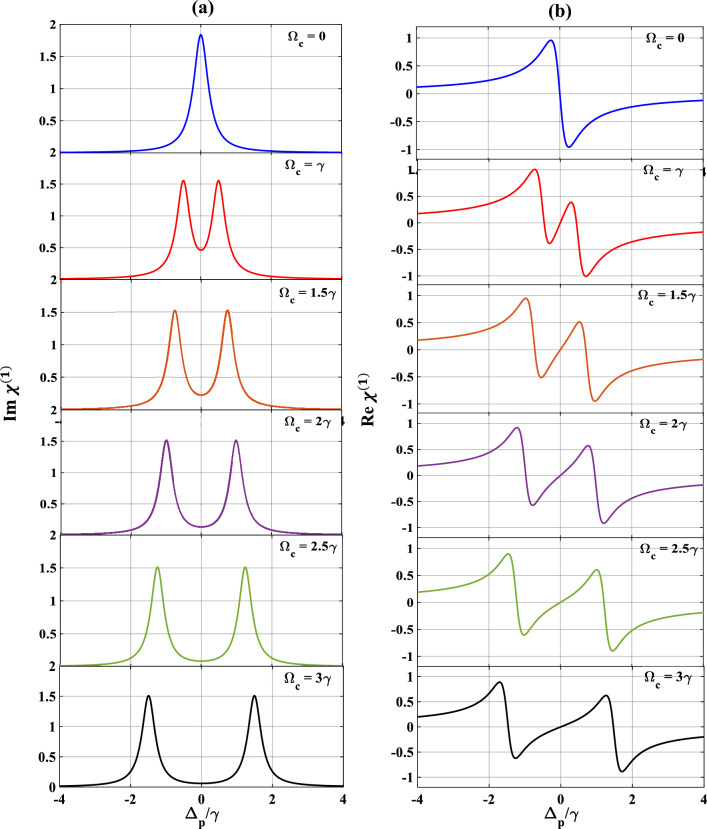
Variation of (**a**) absorption, (**b**) dispersion with normalized probe detuning for different first control field. Here, $\Omega_{c} = 0$ (blue), $\gamma$ (red), $1.5\gamma$ (brown), $2\gamma$ (violet), $2.5\gamma$ (green), and $3\gamma$ (black). Other parameters are $\Omega_{p} = 0.1\gamma , \gamma = 10 \times 10^{12} {\text{s}}^{ - 1}$, $\Omega_{k} = 0, p = 0$ and $\theta = \frac{\pi }{4}$.

Continuing to the study of the absorption and dispersion, at first, we switch ‘off’ the first- and the second-control fields $\left( {\Omega_{c} = \Omega_{k} = 0} \right)$ and tuned the probe field to $\Omega_{p} = 0.1\gamma$ which simulates the features of a normal absorption (subplot a) and dispersion (subplot b) spectrum, as depicted in Fig. 2. The profile obtained in Fig. 2 are free from the effects of SGC parameter, and resembles with the features of a simple two-level system, where an absorption peak and a steep dispersion with negative slope around probe resonance $\left( {\Delta_{p} /\gamma = 0} \right)$ are obtained ($\Omega_{c} = 0;$ blue). Next, we turn ‘on’ only the first control field to $\Omega_{c} = { }\gamma$ (red), keeping the other parameters same as earlier, and depicted the profile of absorption and dispersion in subplots 2(a) and 2(b). Here, we observe the splitting of absorption profile into two peaks producing a transparency window around $\Delta_{p} /\gamma = 0$, which is indeed the signature of EIT window. The dispersion profile thus created also shows a different behaviour by creating a positive slope around $\Delta_{p} /\gamma = 0$, which indicates the change of group velocity from an anomalous to a normal dispersion regime. Now, we keep on increasing the strengths of the first control field to the values $\Omega_{c} = 1.5\gamma$ (brown), $2\gamma$ (violet), $2.5\gamma$ (green), and $3\gamma$ (black), while the second control field is still turned ‘off’. The changes in the absorption and dispersion profiles are depicted in different colours, where we obtain the respective broadening of the EIT window and simultaneous stretching of the positive and negative slopes of the dispersion curve. The results obtained above are analogous to that of a simple three-level ladder type MQW system^51^.

**Figure 3:**
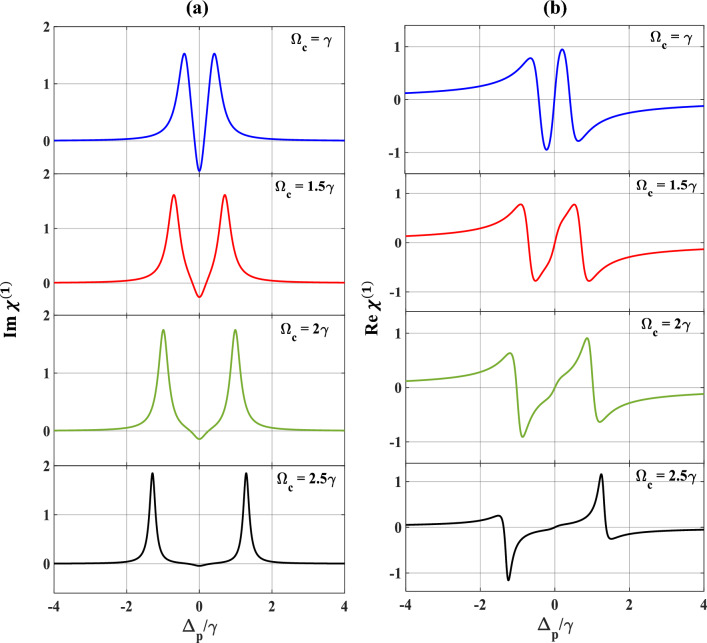
Variation of (**a**) absorption, (**b**) dispersion with normalized probe detuning for different first control field. Here, $\Omega_{c} = \gamma$ (blue), $1.5\gamma$ (red), $2\gamma$ (green), and $2.5\gamma$ (black). Other parameters are $\Omega_{p} = 0.1\gamma$, $\Omega_{k} = 0$, $p = 1$ and $\theta = \frac{\pi }{4}$.

Now, we focus our attention on the study of the optical response in the presence of SGC. In this case, the suitable parameters chosen are, $p = 1,\theta = \frac{\pi }{4}$ and $\Omega_{k} = 0$, and the results are presented in Fig. 3. Quite interestingly, the nature of the absorption and dispersion spectra has changed. For $\Omega_{c} = { }\gamma$ (blue), as evident from subplots 3(a) and 3(b), the transparency region of the absorption spectrum has now been converted into a region of gain-like feature, while the dispersive spectrum displays a small splitting at the region of probe gain. This feature arises due to the imposed SGC conditions on the system, where the SGC term $p$ modulates the system parameters in such a way that it induces some population distribution mechanism resulting in probe gain $\left( {Im \left( {\chi^{\left( 1 \right)} } \right) < 0} \right)$ rather than probe absorption^26^. The interplay of the strong control field and the SGC parameter $p$ results in the reduction of the process of spontaneous emission which manifests in the form of amplification in the medium. Further, an increase in the strengths of the first control field to ${\Omega }_{c}= 1.5\gamma$ (red), and $2\gamma$ (green) results in the broadening and decrease in the probe gain profile, while the dispersive profile shows some small amount of stretching and splitting around the gain region. Finally, at ${\Omega }_{c}=2.5\gamma$ (black), the probe gain feature is lost and the dispersive curve shows small amount of stretching between positive and negative slopes, which are evident from subplots 3(a) and 3(b). Thus, the SGC can be effectively obtained by tuning the strength of the first-control field to suitable values, there by manipulating the absorption through EIT window. The simultaneous modification in the dispersive behaviour opens up the possibility of controlling the light in between anomalous and normal regions which can be applied potentially for optical switching techniques.

### *Effect of *${\Omega }_{k}$* on SGC*

**Figure 4:**
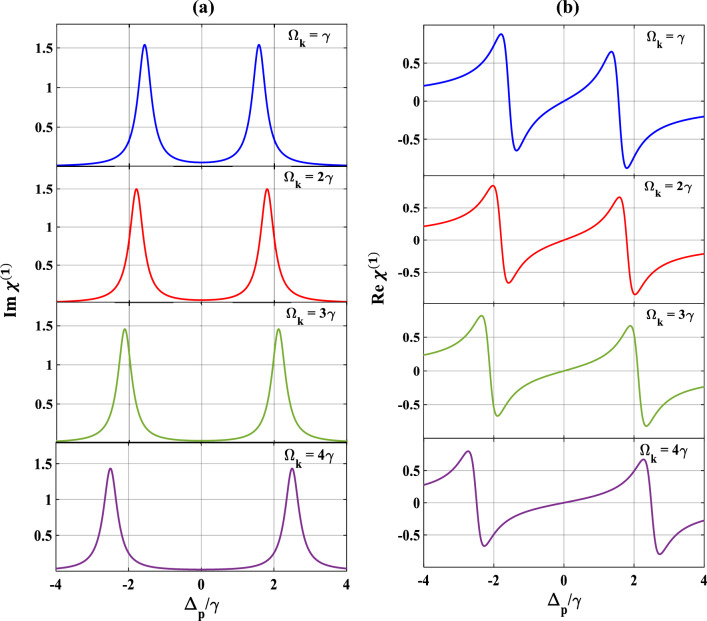
Variation of (**a**) absorption, (**b**) dispersion with normalized probe detuning for different second control field. Here, $\Omega_{k} = \gamma$ (blue), $2\gamma$ (red), $3\gamma$ (green), and $4\gamma$ (violet). Other parameters are $\Omega_{p} = 0.1\gamma$, $p = 0$, $\Omega_{c} = 3\gamma$, and $\theta = \frac{\pi }{4}$.

We now study the optical responses of the MQWs under the effect of the second control field in absence as well as in presence of the SGC parameter. First, we set the strength of the probe and first control field as,$\Omega_{p} = 0.1{ }\gamma$ and $\Omega_{c} = 3{ }\gamma$, respectively, and plot the optical responses in absence of SGC effects, as depicted in Fig. 4. The MQWs then behaves as a combination of the two-ladder type system (or, commonly referred to as the Y-type system) in the presence of the additional control field. From subplots 4 (a) and 4(b), we observe a broadened EIT spectrum along with the spreading of the dispersive profile when the second-control field is set to $\Omega_{k} = { }\gamma$ (blue). With further increase in the value of $\Omega_{k} = 2\gamma$ (red), $3\gamma$(green), and $4{ }\gamma$ (violet), the optical responses result with additional broadening of the EIT window accompanied by spreading of the dispersive (normal dispersion) curve, which are depicted in subplots 4(a) and 4(b). These features are also similar to Fig. 2, only the difference being the auxiliary contribution to the absorption/dispersion spectra due to the second control field leading to more widening of the EIT window and spreading of the dispersion spectrum.

**Figure 5:**
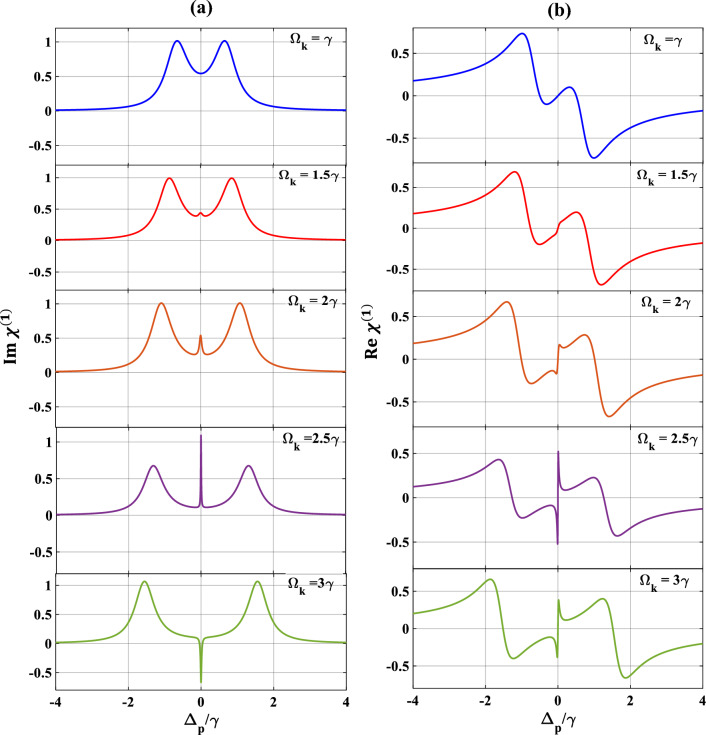
Variation of (**a**) absorption (**b**) dispersion with normalized probe detuning for different second control field. Here, $\Omega_{k} = \gamma$ (blue), $1.5\gamma$ (red), $2\gamma$ (brown), $2.5\gamma$ (violet) and $3\gamma$ (green). Other parameters are $\Omega_{p} = 0.1\gamma$, $p = 1$, $\Omega_{c} = \gamma$ and $\theta = \frac{\pi }{4}$.

**Figure 6:**
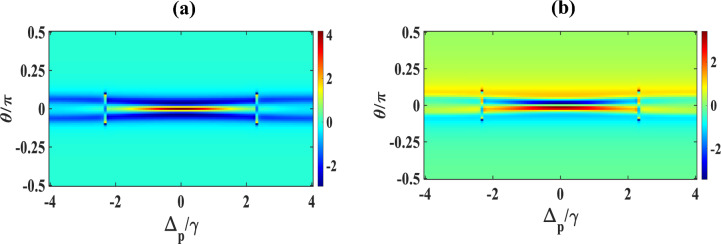
Contour plot of (**a**) absorption, (**b**) dispersion with normalized probe detuning and angle between dipole moment matrix elements. Other parameters are $\Omega_{p} = { }0.1\gamma , \Omega_{c} = \gamma , \Omega_{k} = 0$, $p = 1$.

**Figure 7:**
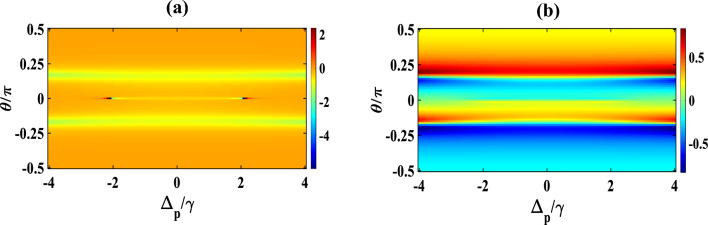
Contour plot of (**a**) absorption, (**b**) dispersion with normalized probe detuning and angle between dipole moment matrix elements. Other parameters are $\Omega_{p} = 0.1\gamma , \Omega_{c} = \gamma , \Omega_{k} = 2.5\gamma$, $p = 1$.

Now, we draw our attention to the characteristics of the absorption/dispersion spectra in presence of SGC as displayed in Fig. 5. We set $\Omega_{c} = \Omega_{k} = \gamma$ while keeping the other parameters unchanged. We observe a similar feature of EIT window and a dispersion spectrum at $\Omega_{k} = \gamma$ (blue) [compare blue colour curve of Fig. 5 with red colour curve of Fig. 2]. With a small increase in the second control field $\Omega_{k} = 1.5\gamma$ (red), the SGC feature appears in the profiles of absorption and dispersion, making a small peak in the middle of the EIT window, and an oscillation in the positive slope of the dispersion at around $\Delta_{p} /\gamma = 0,$ as evidently displayed in Fig. 5b. Further increase in the strengths of $\Omega_{k} = 2\gamma$ (brown), and $2.5\gamma$ (violet), respectively, increases the strength of the SGC and thus increases the splitting of the dispersive spectrum around the SGC region, which are clearly visible from the subplots 5(a) and 5(b). Interestingly, $\Omega_{k} = 3\gamma$ (green), the absorption of the probe shows dip like feature (indicating gain) and dispersion changes from positive to negative within $- 1 \le \Delta_{p} /\gamma \le 1$ with an oscillation around $\Delta_{p} /\gamma = 0$, which signifies the change in group velocity from normal to anomalous. Based on the above results, it can be safely inferred that the control on the optical response improves in a greater extent with the second control field under SGC. In contrast to the three-level system, where the population may get trapped owing to various relaxation processes resulting in the decrease in the overall coherence of the system. In our chosen system, the additional control field in presence of SGC helps in mitigating this trapping process by providing additional decay paths for efficient relaxation and population transfer which increases the quantum interference process and thus the overall coherency of the system. Hence, we may conclude that the presence of both control fields results in efficient tuning and at the same time flexible coherent control of optical responses in the Y-type MQWs via SGC. The opening of the multiple dispersion regions (i.e., positive and negative slopes) allows adequate possibility of performing optical switching actions via SGC in MQWs. In order to get a better visualization of the above features, we illustrate some appropriate contour plots in Figs. 6 and 7, to justify the above figures with suitable strengths of control fields chosen same as that applied for Figs. 3 and 5, respectively. In Fig. 6a, taking $\Omega_{c} = {\upgamma }$, $\Omega_{p} = 0.1{\upgamma }$ and $\Omega_{k} = 0$, we trace the changes in contour of absorption for a range of $\theta /\pi$ with respect to $\Delta_{p} /\gamma$. It is observed that the transparency region (shown by horizontal central red line) gradually changes to gain (shown by the light blue colour extended both sides of the red line) in absence of the second control field ($\Omega_{k} = 0$) under SGC effect. The vertical lines around $\Delta_{p} /\gamma = \pm\space2.9$ are small disruptions towards absorption boundaries. The colour bar in right indicates the magnitude of $Im{ }\left( {{\upchi }^{\left( 1 \right)} } \right)$. Keeping the control parameters same, the contour of the dispersion for a range of $\theta /\pi$ with respect to $\Delta_{p} /\gamma$ is plotted in Fig. 6b, where the colour bar in right indicates the magnitude of $Re{ }\left( {{\upchi }^{\left( 1 \right)} } \right)$. For dispersion contour also, similar argument is valid. Now, we try to understand the contour plot of Fig. 7a by taking an appropriate value of $\Omega_{c} = {\upgamma }$, $\Omega_{p} = 0.1{\upgamma }$ and $\Omega_{k} = 2.5{\upgamma }$ as employed for violet curve of Fig. 5. Here, we observe that in the absorption contour in presence of the second control field ($\Omega_{k} = 2.5{\upgamma })$, the SGC feature is the most prominent (as seen in the central yellow line). Similar feature may also be observed upon examination of Fig. 5 ($\Omega_{k} = 2.5\gamma )$. The corresponding dispersion contour, as depicted in Fig. 7b, toggles between positive and negative slopes as can be seen from the colour bar in the right, which again may be compatible with dispersion feature of Fig. 5 with second control $\Omega_{k} = 2.5\gamma$.

### Effect $\Delta_{c} , \Delta_{k} , \theta ,$ and $p$ on SGC

**Figure 8:**
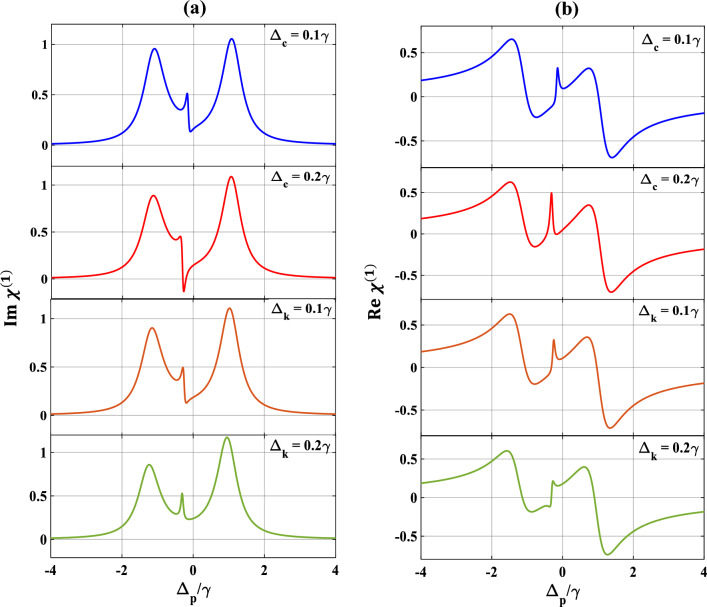
Variation of (**a**) absorption, (**b**) dispersion with normalized probe detuning for different first control detuning as $\Delta_{c} = 0.1\gamma$ (blue), $\Delta_{c} = 0.2\gamma$ (red), and second control detuning as $\Delta_{k} = 0.1\gamma$ (brown), $\Delta_{k} = 0.2\gamma$ (green). Other parameters are $\Omega_{p} = { }0.1\gamma$, $p = 1$, $\Omega_{c} = \gamma$, $\Omega_{k} = 2\gamma$ and $\theta = \frac{\pi }{4}.$ Here, in upper two subplots (denoted as blue and red) $\Delta_{k} = 0,$ lower two subplots (denoted as brown and green) $\Delta_{c} = 0.2\gamma$.

In this subsection, we are mainly investigating the effect of other parameters such as $\Delta_{c} , \Delta_{k} , \theta$ and $p$ which can affect the SGC features in the Y-type system. Figure 8 displays the variation of the absorption/dispersion spectra with $\Delta_{p} /\gamma$ for different first and second control detunings. We set the control detuning to $\Delta_{c} = 0.1\gamma$ (blue) and observe an asymmetry and a slight shift in the position of the SGC feature along with the noticeable shifting in the associated dispersion curve away from the line centre ($\Delta_{p} /\gamma = 0)$ which is clearly visible in subplots 8(a) and 8(b). This asymmetry in the SGC increases along with positional shifting and interestingly absorption is slightly reduced around $\Delta_{p} = - 0.1\gamma$ when $\Delta_{c}$ is tuned to $0.2\gamma$ (red) which is observed in subplot 8(a). Now tuning of $\Delta_{k} = 0.1\gamma$ (brown) and $0.2\gamma$ (green)) also results in asymmetry and shift in the positions of SGC, and the associated dispersion. Therefore, we may safely conclude that the tuning of both the first and second control field detunings serve as an important parameter for the effective control of SGC in MQWs system.

**Figure 9:**
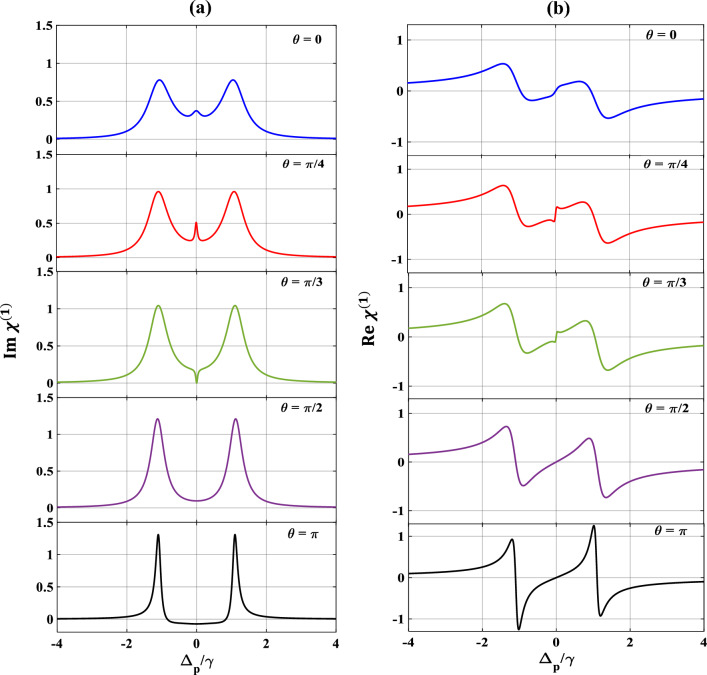
Variation of (**a**) absorption, (**b**) dispersion with normalized probe detuning for different angle between dipole moment matrix elements. Here, $\theta = 0$ (blue), $\frac{\pi }{4}$ (red), $\frac{\pi }{3}$ (green), $\frac{\pi }{2}$ (violet) and $\pi$ (black). Other parameters are $\Omega_{p} = 0.1\gamma$, $p = 1$, $\Omega_{c} = \gamma$ and $\Omega_{k} = 2\gamma$.

**Figure 10:**
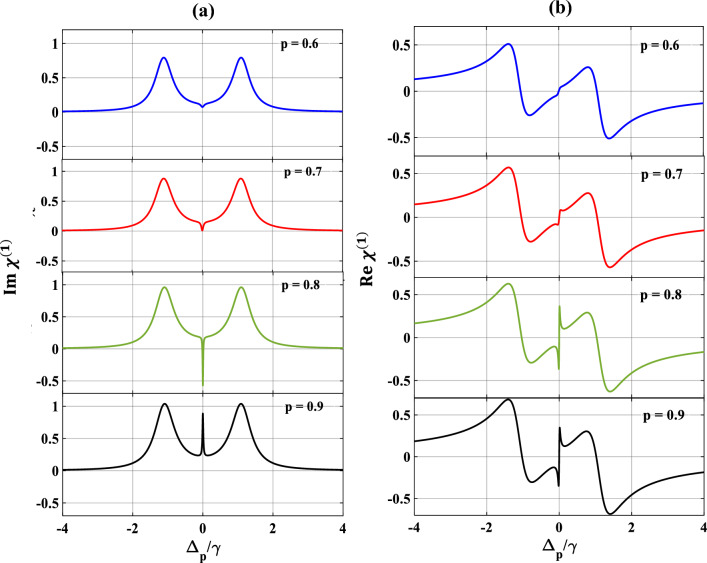
Variation of (**a**) absorption, (**b**) dispersion with normalized probe detuning for different values of SGC parameter ($$p)$$. Here, $$p = 0.6$$ (blue), $$0.7$$ (red), $$0.8$$ (green), $$0.9$$ (black). Other parameters are $$\Omega_{p} = 0.1\gamma$$, $$\Omega_{c} = \gamma$$, $$\Omega_{k} = 2\gamma$$ and $$\theta = \frac{\pi }{4}$$.

We now pay our attention to the control over SGC by manipulating $\theta$ which is the angle between two non-orthogonal dipole moment matrix elements and presented the results in Fig. 9. Here, we vary $\theta$ from $0$ to $\pi$, selecting other parameters as $\Omega_{c} = { }\gamma$, $\Omega_{k} = { }2\gamma$, $p = 1$ and $\Delta_{c} = 0.$ From subplots 9(a) and 9(b) it is seen that, when $\theta = 0$ (blue), optical response of the probe shows the creation of SGC within the EIT window along with splitting of the dispersion curve. Interestingly, with the increase in the value of $\theta$ to $\frac{\pi }{4}$ (red), the strength of the SGC also increases and inverts at $\theta = \frac{\pi }{3}$(green) as shown in subplot 9(a). Finally, at $\theta = \frac{\pi }{2}$ (violet), the SGC feature is completely lost. With further increase of $\theta$ at $\pi$ (black), the profile displays sharp amplifications of the probe field along with steep positive dispersion between $- \gamma$ and $\gamma$ of the probe detuning. Similarly, we also vary the SGC parameter $p$ from $0.6$ to $0.9$ which results in similar features as exhibited in Fig. 10. For $p=0.6$ (blue), there creates a small dip in absorption and a steep in dispersion profiles, respectively. These SGC features are increased more for $p=0.7$ (red). For $p=0.8$ (green), the dip in absorption gets more deepen, and the oscillation in dispersion is more pronounced. For $p=0.9$ (black), the SGC effect creates a peak at the middle of the absorption profile, thereby splitting into two transparency windows, whereas, the oscillation in dispersion is almost similar as before. Thus, we can conclude that the control over SGC is dependent upon the proper choice of the angle between two non-orthogonal dipole moment matrix elements ($\theta$) and the SGC parameter ($p$).

Before closing, it would be worth mentioning that in the currently existing literature, there seems to be a fundamental lack of understanding regarding the tunability of quantum system parameters^52,53^ as there exists a difference between the theoretical models and the realistic quantum system^54^. In this investigation, we have considered a theoretical model for the study of SGC under EIT regime within the MQWs, where the information on the system parameters like dipole moments, dephasing time and laser field strengths are expected to provide important insights in understanding the associated coherence effects. By studying the SGC behaviour under EIT in MQWs with fixed parameters, it is anticipated that the researchers can gain an understanding on the limitations and challenges associated with the actual experimental realization in quantum systems. Also, the theoretical predictions and experimental results can be compared in order to provide a basis for validity and applicability of our model. The tuning parameters employed here are taken from realistic laser field parameters and our model may be experimentally realizable in near future. Further, one may tune the model parameters and vary them to cause interesting effects by coupling them with electron transport models in order to gain a more realistic experimental perspective on the application of EIT effect^44–46^. Thus, it is expected that our model will establish a bridge between theory and experiment, providing a more comprehensive understanding of quantum phenomena and their potential applications in optoelectronic devices.

## Conclusion

The above analysis of the four-level Y-type system in MQWs quite successfully demonstrates the potential of harnessing SGC to exert precise control over the behaviour of transparency and dispersion. We see that the coherent phenomenon of SGC introduces a new dimension to the manipulation of optical properties enabling the researchers to tailor the resonant characteristics of the system. We observe that in absence of SGC and in presence of a first control field, a single narrow transparency window accompanied by a positive slope (normal dispersion) around the EIT region is obtained. In presence of SGC, the transparency window is converted into an amplification window, while the dispersion window shows some small amount of splitting in the amplification regime. With the increase in the strengths of the first control field, there occurs a reduction in the amplification window followed by stretching in the slopes of the dispersion curves. Interestingly, by tuning the second control field in presence of SGC, a small noticeable peak can be observed which is the characteristic feature of SGC. It is noticed that the amplitude of the SGC peak could be controlled by tuning the control fields strength. Also, the position of the SGC peaks could be shifted by manipulating the control field detuning of the first and second control fields and also by varying the SGC parameter ($p$). Furthermore, our findings highlight the inherent advantages of SGC in manipulating the dispersive behaviour of the medium favourable to applications based on light propagation. It is important to discuss a fundamental aspect of light-matter interaction here which is the uncertainty principle. Since the wavelengths of the three lasers (one probe and two control lasers) differ, it implies a significant difference in the net coherent superposition of the states associated with the wavelengths of these lasers. This in general implies a broad range of momentum (or wavelength) values contributing to the overall state which in turn leads to a loss of coherence as the different components of momentum interfere less effectively with each other due to their phase differences. In experiments involving quantum coherent phenomena such as interference, EIT and SGC maintaining the coherence typically requires controlling and matching the wavelengths of the involved laser fields. Thus, the uncertainty principle implies an inherent trade-off between the precision of knowing certain physical properties, such as position and momentum (or wavelength), which can impact the coherence of such quantum well systems.

The comprehensive investigation carried out in this article reveals that the coherent interplay induced by SGC offers a promising avenue for enhancing light-matter interactions in the MQWs. This study is expected not only to contribute to our fundamental understanding of SGC in MQWs but also to open new frontiers in quantum communications, computation, and photonics pertaining to quantum technology.

## Data Availability

The datasets used and/or analysed during the current study available from the corresponding author on reasonable request.
